# Modeling and Assessing the Spatial and Vertical Distributions of Potentially Toxic Elements in Soil and How the Concentrations Differ

**DOI:** 10.3390/toxics9080181

**Published:** 2021-07-31

**Authors:** Samuel Kudjo Ahado, Chukwudi Nwaogu, Vincent Yaw Oppong Sarkodie, Luboš Borůvka

**Affiliations:** 1 Department of Soil Science and Soil Protection, Faculty of Agrobiology, Food and Natural Resources, Czech University of Life Sciences Prague, Kamýcká 129, 16500 Prague, Czech Republic; ahados@af.czu.cz (S.K.A.); oppong_sarkodie@af.czu.cz (V.Y.O.S.); boruvka@af.czu.cz (L.B.); 2 Department of Environmental Management, Federal University of Technology, Owerri, P.M.B. 1526, Owerri 460114, Nigeria; 3 Department of Forest Protection and Entomology, Faculty of Forestry and Wood Sciences, Czech University of Life Sciences Prague, Kamýcká 129, 16500 Prague, Czech Republic

**Keywords:** heavy metals, positive matrix factorization, contamination factor, pollution load index, GIS-kriging

## Abstract

A healthy soil is a healthy ecosystem because humans, animals, plants, and water highly depend upon it. Soil pollution by potentially toxic elements (PTEs) is a serious concern for humankind. The study is aimed at (i) assessing the concentrations of PTEs in soils under a long-term heavily industrialized region for coal and textiles, (ii) modeling and mapping the spatial and vertical distributions of PTEs using a GIS-based ordinary kriging technique, and (iii) identifying the possible sources of these PTEs in the Jizerské Mountains (Jizera Mts.) using a positive matrix factorization (PMF) model. Four hundred and forty-two (442) soil samples were analyzed by applying the aqua regia method. To assess the PTE contents, the level of pollution, and the distribution pattern in soil, the contamination factor (CF) and the pollution load index load (PLI) were applied. ArcGIS-based ordinary kriging interpolation was used for the spatial analysis of PTEs. The results of the analysis revealed that the variation in the coefficient (CV) of PTEs in the organic soil was highest in Cr (96.36%), followed by Cu (54.94%) and Pb (49.40%). On the other hand, the mineral soil had Cu (96.88%), Cr (66.70%), and Pb (64.48%) as the highest in CV. The PTEs in both the organic soil and the mineral soil revealed a high heterogeneous variability. Though the study area lies within the “Black Triangle”, which is a historic industrial site in Central Europe, this result did not show a substantial influence of the contamination of PTEs in the area. In spite of the rate of pollution in this area being very low based on the findings, there may be a need for intermittent assessment of the soil. This helps to curtail any excessive accumulation and escalation in future. The results may serve as baseline information for pollution assessment. It might support policy-developers in sustainable farming and forestry for the health of an ecosystem towards food security, forest safety, as well as animal and human welfare.

## 1. Introduction

Soil is an indispensable component of an ecosystem that directly or indirectly links and maintains the Earth’s four spheres (namely the lithosphere, biosphere, hydrosphere, and atmosphere). However, this essential potential of soil has, in recent times, been threatened by heavy metals or potentially toxic elements (PTEs). Interestingly, the chemistry of soil makes it vulnerable to high concentrations of heavy metals or PTEs. At a required concentration, most PTEs such as Cr, Cu, Fe, Mn, Zn, Ni, Mo, Co, Se, and others are essential for plants, animals, or humans [1,2]. The presence of PTEs in soil has been attracting reasonable attention because of their ecological and biological risks. Several studies in different biomes have been performed to identify the sources of PTEs in the soils [3,4,5,6,7,8,9,10,11,12,13,14,15,16].

Natural phenomena and anthropogenic activities are the two major sources that determine the concentrations of PTEs in soils [17,18,19,20,21,22,23,24]. Natural phenomena are described as the components generated from parent material, whereas anthropogenic sources primarily originate from acute human activities [17,18,25,26]. Many authors have revealed that natural sources of some PTEs (such as Pb, Cd, and Hg) have been surpassed by anthropogenic deposits into soils due to pedogenesis [17]. Industrial inputs, the combustion of fossil fuels, municipal wastewaters, and sewage sludge have been identified as anthropogenic sources of metals [11,14,27,28]. Furthermore, intensive agricultural practices have been reported to increase PTEs in soils [16,17]. In addition, automobile and vehicle emissions, road dusts, and military activities also account for increases in PTEs [4,29]. It has been estimated by some authors that agricultural practices contributed to 79.6%, 56%, and 63% of the annual concentrations of Cu, Zn, and Cd, respectively [30]. The authors further summarized that the total annual input of Pb (85%), Ni (67.5%), and Cr (43%) found in soil emanates from industrial atmospheric deposition.

The safety of plants, food, animals, and human health have been threatened by the accumulation of PTEs in soil. Toxic elements are discharged into the soil and subsequently absorbed by plants, which are consumed by animals and humans [12]. In some cases, the PTEs penetrate into surface and underground water, which are used by living organisms including humans [31,32,33].

In the Czech Republic, edible mushrooms grown in a smelting area were reported to have been contaminated by the atmospheric deposition of Pb [34]. In Germany, there has been an urgent call to address the Pb contents in plant-based foodstuffs including bread and potatoes, which are important suppliers of this metal in human meals [35]. The yearly deposition of Cr to soils in the UK was 327 tons [36]. In addition, the study reported that 126 tons out of the 327 tons were emanated from chemical fertilizers (mostly phosphate), while 83 tons originated from atmospheric deposition and 78 tons came from sewage sludge [36]. The effect of Cr is not only recorded in food crops but also in forest trees. The health of forest plants has been at risk because of exacerbated Cr content in the soil [37]. Globally, there have been reports on the effects of increased Cu, Fe, Mn, Mo, Zn, Ni, and other PTEs on soils, plants, animals, humans, and water. Therefore, the issue has become of critical concern to the government and the stakeholders, including decision makers. 

The urgency of the situation demands a robust assessment with effective quantitative and qualitative analyses. An investigation of the PTEs in soil and their sources is the principal purpose for preserving and enhancing soil quality in most areas in the world. Thus, to develop reliable policies for a sustainable soil safety for an area, it is important to have good information on the soil and its contamination level. In recent years, several analyses including statistical, geostatistical, geo-accumulation index, multivariate and modeling, as well as potential ecological risk index analyses have been proposed and applied to investigate the source, degree, and spatiotemporal state of PTE pollution in the soil [3,4,5,6,12,14,24,28,29,31,32,33]. As reported by some authors, an assessment of the contents and distribution of PTEs in soil requires intensive and robust sampling to investigate the soil conditions under distinct soil types [38,39,40,41,42]. Furthermore, considering the high temporal, spatial, and vertical variability in the uppermost soil layers of a forest, a substantial number of samples need to be examined in order to thoroughly quantify the soil adequately along an extensive scale [43]. Routinely, the study of PTE content in soil has been performed following the regular laboratory chemical methods, including atomic absorption or inductively coupled plasma analysis. These methods are expensive and time-consuming and involve consecutive serial procedures with growing complications [44,45]. Thus, a systematically structured and affordable analytical method to monitor and assess the PTEs in soil on an appropriate vertical and spatial scale is necessary [46], especially when a tangible number of sampling points are considered. The flexibility and rapid accessibility of the positive matrix factorization (PMF) model in assessing soil pollutions is remarkable [6,41]. This analytical method has high functionality for the investigation of the PTEs in soil. The PMF provides a great advantage in detecting and monitoring PTEs in organic and mineral soils: it is one of the best and latest models [6,41,47,48].

The study area is located in a northern part of the Czech Republic. The area is called Jizerské hory Mountains (Jizera Mts.). The area was polluted by past accumulation of PTEs from human and natural sources. However, there have been ongoing policies and efforts by the government and the people to ameliorate the problem, yet the impact is still prevailing in the ecosystem (mainly in the soil and vegetation). This is partly because, after soil is polluted, it takes a longer period for remediation and for ecosystem recovery processes to be completed [49]. Second, a large amount of the PTEs are enriched in the acidified forest soil, and these elements are still being discharged [5,50,51]. Some authors have reported health risks from the PTEs in high-altitude mountains in Europe including the Jizera Mts. [52,53,54]. For example, in a study performed by EscartÍn and Porte [52], the authors reported that a high percentage (76%) of polycyclic aromatic hydrocarbon (PAH) metabolites were detected in trout from the Central European high Mountains lake and that this has a high health risk. There have been many studies that focused on the acidification by sulfur and nitrogen oxides in the area. Studies focusing on the spatial and vertical distributions as well as the content and hotspots of PTEs are crucial for closing the gap in sustainable pollution assessment in the area. The benefits of applying PMF to investigate PTEs in the soil is commendable [55]. This study aims (i) to assess the concentrations of PTEs in the soil under Jizera Mts. in the Liberec region of Czech Republic after long-term, heavy industrialization; (ii) to model and map the spatial and vertical distributions of the PTEs using a GIS-based ordinary kriging technique; and (iii) to identify the possible sources of these PTEs and their contamination levels in the area using a PMF model. The findings from this study may serve as a baseline for the pollution assessment of farmland and forest soil quality in the Czech Republic and in Europe. The results might support policy-developers in sustainable farming and forestry for the health of the ecosystem and for food security, forest safety, as well as animal and human welfare.

## 2. Materials and Methods

### 2.1. Study Area

**Figure 1:**
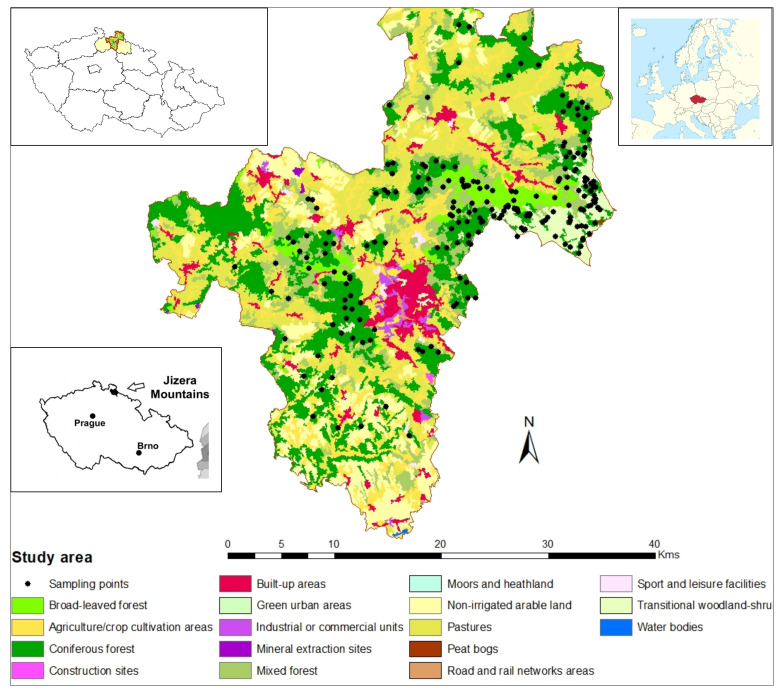
Sampling points and land use–land cover of the Jizera Mts. area derived from the CORINE database (**central** Map), the location of the Jizera Mts. in Liberec region in the northern part of the Czech Republic (**top left** and **down left** maps), and the location of the Czech Republic in Europe (**top right** map).

The study covered about 110 square kilometers in the Jizera Mts. The height above sea level of the area ranges from 600 to 1100 m. The average yearly temperature falls between 3 and 6 °C, which is contingent upon the altitude. The annual precipitation reaches about 1500 mm at the top of the mountains. Most areas are covered by forests (Figure 1), though in some areas, the regeneration of trees has been slow after intensive forest decline in the 1980s and 1990s [5,13]. Coniferous species, namely Norway spruce (*Picea abies*) and the European beech (*Fagus sylvatica*), are key forest trees. There are also areas with pockets of peatbogs. PTE pollution in the area is considered to have been emanated from atmospheric deposition released from the coal, textile, and steel industries and from agricultural activities. Geologically, the area is characterized by principal acidic bedrocks such as granite (granodiorite) and gneiss. Haplic/Entic Podzols, Stagnosols, and Cambisols are the predominant soils [56,57,58]. In most of the area, especially in the higher altitudes, the mor form of humus dominates while the moder humus type is observable only at lower altitudes [59]. The value of the soil pH was relatively low (Table 1), thus contributing to the high acidic condition of the area.

### 2.2. Soil Sampling and Laboratory Analysis 

At every 3 km, soil samples were collected for both organic soil and mineral soil (to the depth of 30 cm). The samples were collected in 3 replicates for each sampling point, and the average value of the sampled points was used for the analysis. The sampling points were located using a handheld GPS system, while samples were collected using either a push probe or bucket auger depending on the terrain. A total of 221 samples each were collected from organic soil (org) and mineral soil (A) across the study area. The collected soil samples were stored in well-labelled plastic bags and taken to the laboratory. The collected samples were air-dried, ground, and sieved with a mesh of size 2.0 mm to obtain a pulverized sample. 

#### Chemical Analysis and Instrument

The presence of elements such as Cr, Cu, Pb, Mn, and Fe in the soil were extracted using the aqua regia standard method (ISO 11466:1995, 1995) to determine their pseudo-total content [60]. For quality control (QC) of the method, the standard addition technique was adopted. For example, the QC of the concentration determination was guaranteed using the SRM 2711 (Montana II soil) reference material (National Institute of Standards and Technology, Gaithersburg, MD, USA). The values achieved were consistent with the reference data. The recovery differences were generally < 10% (*n* = 3). The detection limits for the elements based on the applied method were as follows: Cr (0.03 mg L^−1^), Cu (0.015 mg L^−1^), Pb (0.05 mg L^−1^), Mn (0.05 mg L^−1^), and Fe (0.15 mg L^−1^). The presence of Fe and Mn in the soils were also investigated; their concentrations posed no threat in the area because their concentrations were far below the EU and world recommended limits.

### 2.3. Contamination Level Analysis for PTEs

The PTE pollution status of the study area was assessed through various contamination assessment indices, namely the contamination factor (CF) and the pollution index (PLI). 

#### 2.3.1. Contamination Factor 

CF is defined as the ratio of metal content in the sample to the background value of the same metal. It is given by the following:

CF = C (metal) Sample/C (metal) background value(1)

where C (metal) is the concentration of metal analyzed from sampled soil and where C (metal) background value is the geochemical background value (or concentration) of that metal.

It is important to state here that the baseline values used were the world values [10]. 

#### 2.3.2. Pollution Load Index (PLI)

The PLI is an estimation and was first proposed by [60]. The pollution load index has been in use for the detection of pollution. It is robust and effective in the comparison of pollution levels in space and time. The PLI was calculated based on the concentration factor of each PTE by focusing on the background value in the soil, where CF is the contamination factor earlier stated (Equation (1)) and the letter ‘n’ signifies the number of metals studied. A pollution load index less than 1 indicates the optimal soil quality, and a PLI that is equal to 1 proves that only the baseline levels of contaminants are present, while a PLI greater than 1 infers the degradation of the quality of the site by [61]. 

The pollution load index (PLI) equation is given by the following:

PLI = n√ (CF_1_ × CF_2_ × CF_3_ × … × CF_n_) (2)

where CF is the contamination factor derived for each metal and where n is the number of metals.

### 2.4. Source Apportionment Using a Positive Matrix Factorization (PMF) Model

The positive matrix factorization (PMF) model is an effective method acquired from the software EPA-PMF v 5.0, Washington DC, USA [55]. It was applied to determine the contribution of PTE sources to contamination in the study area. The mathematical method is a receptor model used in calculating the contribution of the sources to samples built on the composition or fingerprints of the sources. The PMF model apportions the collaborations of elements in soil composition by solving chemical mass balance:

(3) $$C_{ij}=\overset{p}{\underset{K=1}{\sum^{\;}}}G_{ik}+F_{kj}+E_{ij}$$

where *C_ij_* signifies the content of PTEs *j* in soil sample I, *p* represents the number of factors (i.e., pollution sources), *G_ik_* shows contribution of factor *k* to soil sample I, *F_kj_* denotes the content of PTEs *j* in factor *k*, and *E_ij_* stands for the residual. 

Additional information on the procedures, methods, and formulas used in this study for determining the soil or site contamination level through the PMF model was followed as specified by [55,62] and as applied by [3,6].

### 2.5. Statistical Analysis and Spatial Modeling 

Basic statistical parameters (such as mean, median, minimum, maximum, standard deviation, and coefficient of variance) were first calculated for each soil property based on horizon. Positive matrix factorization (PMF, EPA version 5.0, Washington, DC, USA) was used for the estimation of source apportionment and contamination level of the PTEs. To determine the relationship between the PTEs in organic and mineral soils, an ANOVA and correlation analysis were used. Ordinary kriging interpolation was used in determining the differences and/or similarities among sites with a proportional distance among them. The interpolation technique enhanced the creation of the spatial distribution maps of the PTEs of the study area. ArcGIS, version 10.7.1, CA, USA [63], was used for processing and visualizing of the spatial data. By applying the ordinary kriging technique, maps of the spatial distribution of these soil properties were generated [64]. The result was validated using the mean error [65,66]. In other words, to determine the accuracy of the produced maps, the mean error (ME) was used for the validation. The formula is shown below in Equation (4):

(4) $$\mathrm{ME}=\sum_{i=1}^{n}\left(x_{1.i}-x_{2.j}\right)/n$$

where x_1_ is prediction of the variable x, x_2_ is measure of that variable, and n is number of records.

## 3. Results and Discussion 

### 3.1. General Description of PTEs Concentrations and Their Spatial Distribution in the Soil

The basic statistical characteristics of the studied PTEs including Cr, Cu, Fe, Mn, and Pb for the organic soil and mineral soil have been described in Table 2. The coefficient of variation (CV) defines the degree of variations within PTE concentrations [67]. A coefficient of variation value less than 20% represents low variability, and a CV that falls between 21–50% is regarded as moderate variability. On the other hand, a CV ranging from 50–100% signifies high variability, while a CV greater than 1 (that is >100%) is described as having extremely high heterogeneity. In this study, the CV of the PTEs in the organic soil increased in the following order: Fe < Pb < Cu < Cr < Mn, accounting for 46.31%, 49.40%, 54.94%, 96.36%, and 97.06%, respectively.

The CV of the PTEs for mineral soil was also in ascending order: Fe (60.12%) < Pb (64.48%) < Cr (66.70%) < Mn (81.9%) < Cu (96.88%). The results derived from the CV revealed a high variability between the PTEs in the mineral soil. Similarly, in the organic soil, the CV for Cr, Mn, and Cu indicated a high variability (Table 2). In general, both the organic soil and the mineral soil revealed high heterogeneity (or variability). All of the PTEs showed relatively high variability in both soil horizons except for the Fe (46.31%) in the organic soil. The spatial distribution of the heterogeneity of the PTEs suggest that the metals are enriched by intensive sources of from the industrial, commercial, domestic and agricultural sectors [3,5,13]. However, the content of the PTEs varies between the soil horizons, yet the organic soil had higher mean values across the metals, excluding Mn. The content of manganese was 18.4 mg kg^−1^ higher in the mineral soil compared with its content in the organic soil. This finding agrees with a report by other authors in the same region [21]. Studies have shown that, in addition to human activities and their associated soil acidifications [13], the geological bedrock of the area also contributes to accruing PTE concentrations [15]. The study area has Podzols and Dystric Cambisols as the prevailing soils [5], and this might have contributed to the high contents of Pb, Fe, and Mn. The concentration of Fe in the study area is remarkable when compared with other metals. This could be attributed to the high acidic soil status of the area (Table 1). As has been earlier reported, the concentration of Fe in the soil solution at optimal soil pH falls between 30 and 550 μg L^−1^, but in high acid soils, it may exceed 2000 μg L^−1^ [68]. Higher concentrations of the PTEs were found in this study area relative to the neighboring regions in the country [3]. In comparison with the European value [10], the world value [10], and the Crati Basin value [69], the contents of Pb in both the organic soil and the mineral soil were higher. The exceptional content of Pb in the study area might be attributed to past intensive anthropogenic activities and the prevailing geological formation of Cambisols. Lead has been reported to exhibit the highest content in a Cambisols soil group [10]. 

### 3.2. Relationships among the PTE Concentrations in the Organic and Mineral Soils

The correlation analysis for the PTE concentrations in the organic and mineral soils revealed that the content of Cr in the mineral soil (Cr_tot_A) showed a significant and strong positive correlation with Cu, Fe, Mn, and Pb in the mineral soil (Table 3). Chromium has a strong relationship with other elements because it is easily mobilized in acidic soils and our study area is highly acidic [5,10]. Furthermore, in the mineral soil, Pb is significantly correlated with Cu and Mn. In the organic soil, Pb has a significant and strong positive relation with Cu and Fe. The strong relation between Pb, Fe, and Mn was documented earlier [10]. The concentrations of Pb in Fe–Mn nodules can be as high as 20,000 mg kg^−1^ [70]. Most of the negative correlations between the PTEs occurred in the inter-horizon and not within the same horizon. The correlation between the elements in the same soil horizon showed more positive relationships. This could be described by the likelihood that they shared the same origin. Furthermore, the correlation of the PTEs in the mineral soil revealed stronger relationships when compared with the correlation in the organic soil. This probably proved that these PTEs are more closely associated in the mineral soil relative to the organic soil. This finding was consistent with a recent report by other authors on the same issue [3].

The ANOVA in Table 4 was used to analyze the distribution of PTE contents in relation to the organic soil and the mineral soil horizons. It was revealed that all of the elements, with the exception Mn, showed significant relationships in both the organic soil and the mineral soil. There have been few studies within and outside the study area that focused on the relationship between soil horizon [8,21]. Consistent with our study, many authors have reported a significant relationship between soil horizon, elevation, and the concentrations of metals. For example, in the Suxian district of Chenzhou City in Hunan Province of China, it was revealed that heavy metal concentrations decreased at low elevation but increased considerably with increasing elevations [8]. Other studies have affirmed that fine-particle metals including Cr and Cu accumulate more at lower elevations [11].

**Figure 2:**
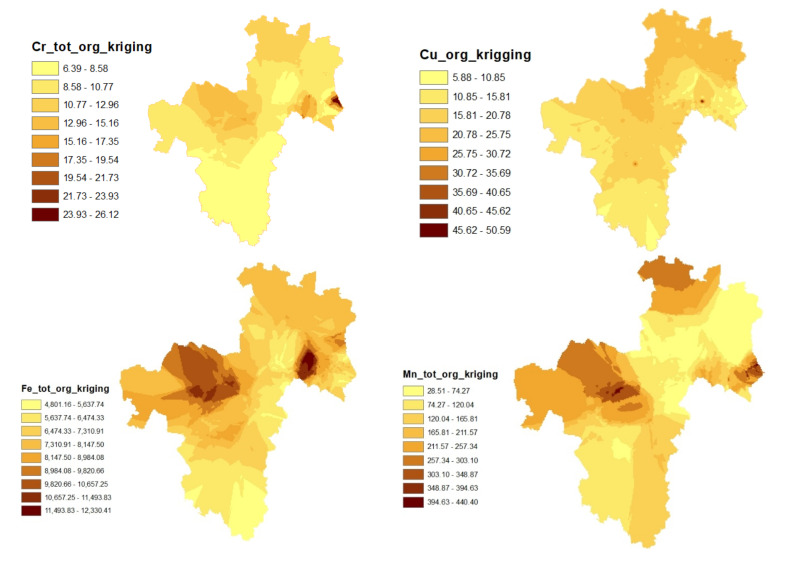

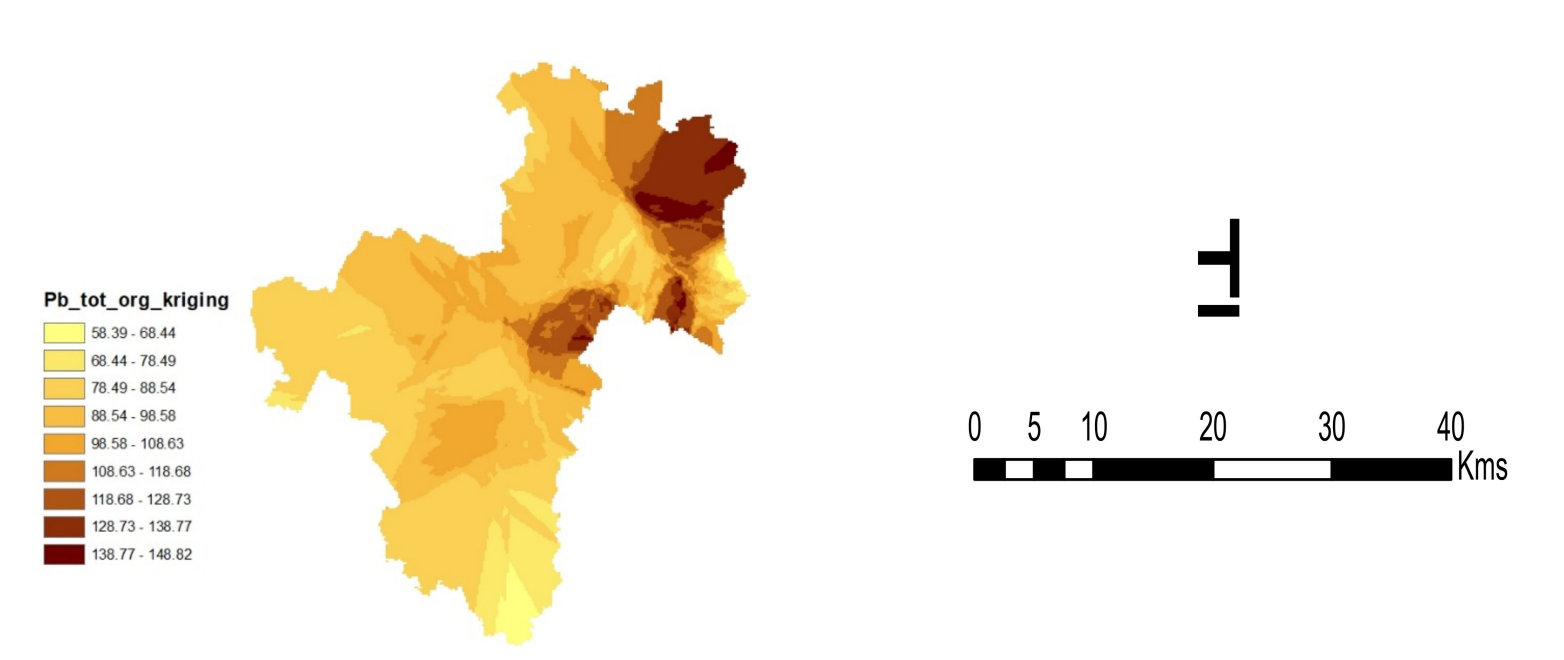
Spatial and vertical distributions of soil characteristics in the organic soil (org) assessed using ordinary kriging (all of the elements are reported in mg kg^−1^). Though Fe and Mn posed no threat in the region, they were mapped/modeled to draw inferences on Cr, Cu, and Pb. In other words, the study attempted to assess if the presence of Fe and Mn in the soil influenced the vertical and spatial distributions of the three other PTEs (namely Cr, Cu, and Pb) in the different soil horizons.

In the organic soil, the highest contents of Cr, Pb, Fe, and Mn were found in the northern and central parts of the area (Figure 2). Studies have revealed a close association between Mn and Fe. Manganese is described as a member of the iron family, and both elements are closely linked in geochemical processes [10]. The author further stressed that Mn cycles follow Fe cycles in various terrestrial environments. Copper on the other hand had a concentration hotspot that spread from the northeast to the northwest. 

**Figure 3:**
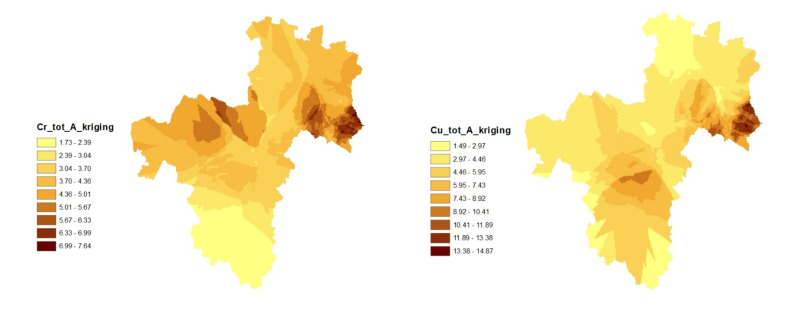

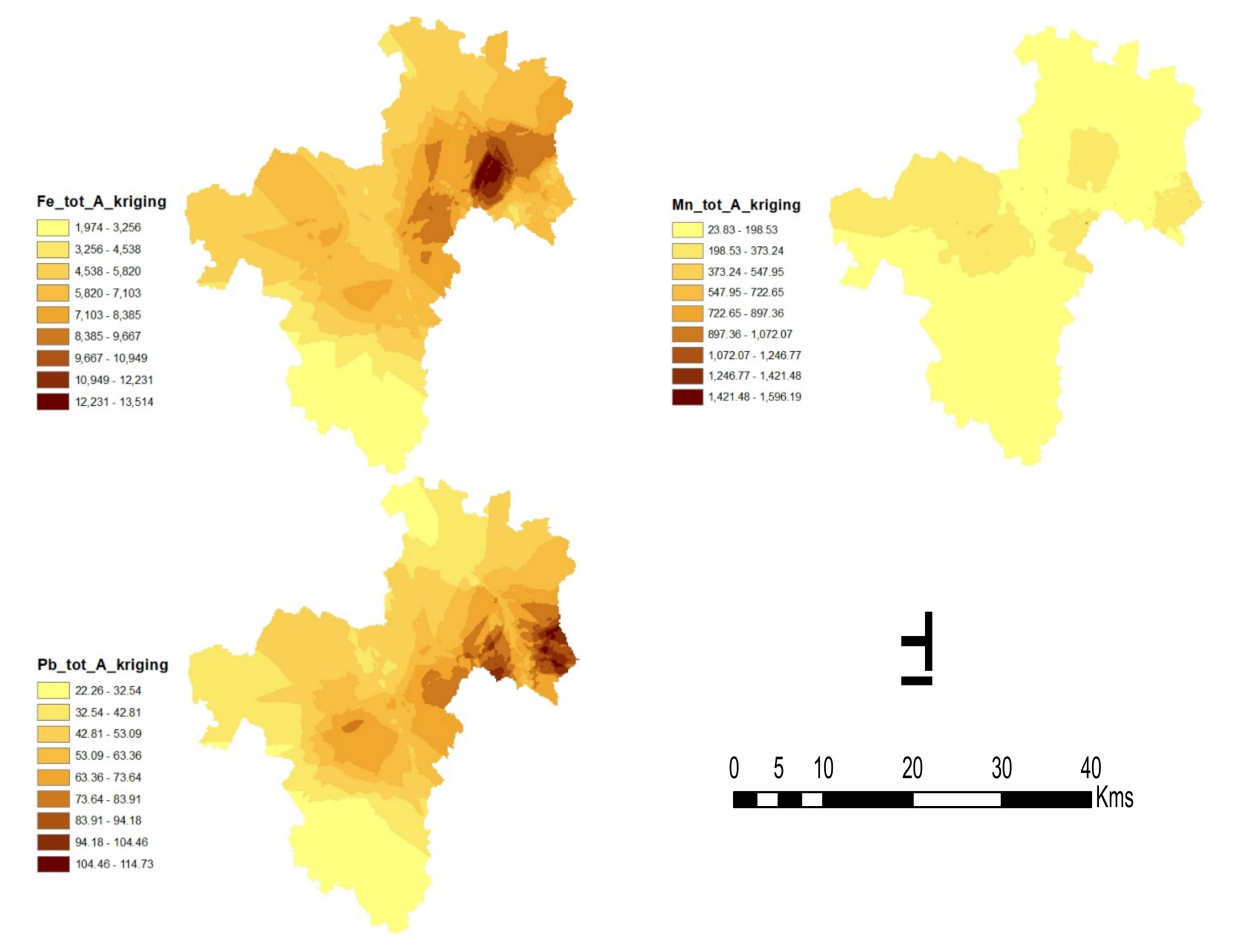
Spatial and vertical distributions of soil characteristics in the surface mineral soil (A) made by ordinary kriging. (all of the elements are in mg kg^−1^). Though Fe and Mn posed no threat in the region, they were mapped/modeled to draw inferences on Cr, Cu, and Pb. In other words, the study attempted to assess if the presence of Fe and Mn in the soil influenced the vertical and spatial distribution of the other three PTEs (namely Cr, Cu, and Pb) in the different soil horizons.

The kriged map of Cu and Pb distributions showed almost the same pattern in the mineral soil (Figure 3). They tend to have higher concentrations towards the east in this area. In this mineral soil, Fe and Cr showed extensive spatial distribution patterns that spread from the northeast, through the central region, and to the northwestern part of the mapped area. On the other hand, the kriged map of Mn distribution showed higher concentrations within the northwest and north-central parts of the area. In general, for both organic and mineral soils, the northern and central parts of the kriged maps revealed more distribution of the elements when compared with the southern part. This could be explained by the historical distribution pattern of industrial and agricultural activities in the study area, which were mainly located in the northern and central parts [71].

### 3.3. Source Apportionment by the Positive Matrix Factorization (PMF) Model

**Figure 4:**
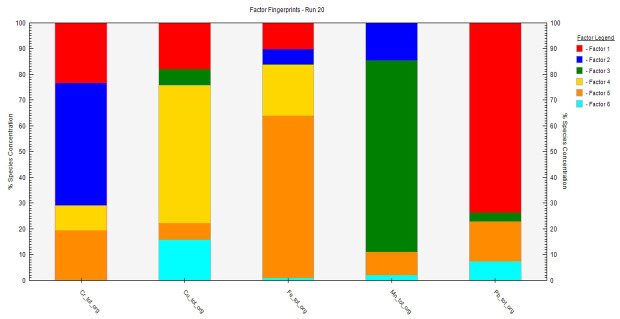
Source fingerprint of the total organic soil (tot-org) from the PMF model analysis showing the percentage contribution of PTEs. Note that the source and availability of Fe and Mn posed no risk to the soils.

**Figure 5:**
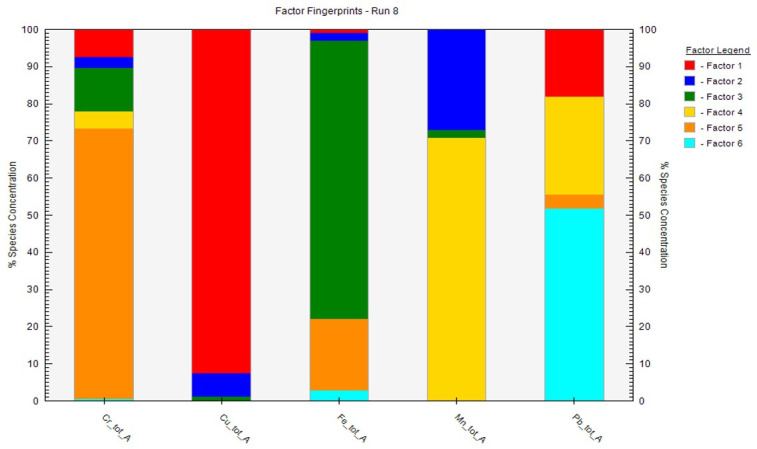
Source fingerprint of the total mineral soil (tot-A) from the PMF model analysis showing the percentage contribution of PTEs. Note that the source and availability of Fe and Mn posed no risk to the soils.

There have been many reports and studies that affirmed that our study area is located within the vicinity of various anthropogenic activities including mining, intensive agriculture, automobile gas emissions, and acute biological sludge, which might affect the soil [4,52,53,72,73]. It is important to examine the sources of PTEs in the study area. Therefore, the PMF model was adopted as one of the best and latest models with high functionalities for effective PTE source identification [6]. The validity and reliability of the analysis are based on minimum Q to model the residual matrix that influences a substantial number of variables. To derive the best result, the PMF model was run 20 times, while the best outputs (which were Run 8 and Run 20) were selected following the software developer’s guide [55]. The PMF analysis produced six factors (see Figure 4 and Figure 5) and disclosed the origin of contributions based on each PTE [62].

In the organic soil, factor 1 was dominated by Pb and Cr, with a factor loading of 73.5% and 23.7%, while in the mineral soil, Cu and Pb had factor loads of 92.2% and 18.3%, respectively (Figure 4 and Figure 5). Factor 2 was characterized by Cr and Mn, and these accrued factor loadings of 45.1% and 15.1%, respectively, in the organic soil layer. On the other hand, in the mineral soil, Mn and Cu accumulated factor loadings of 28.3% and 6.7%, respectively. The factor 3 load consisted mainly of Mn (72.6%) and Cu (4.9%) in the organic soil, while Fe (64.8%) and Cr (11.9%) accrued in the mineral soil. Furthermore, factor 4 load was characterized by Cu (51.7%) and Fe (18.3%) in the organic soil while Mn (71.1) and Pb (26.4%) accumulated in the mineral soil. The factor 5 had Fe (42.5%), Cr (19.6%), and Pb (14.2%), accounting for the highest elements in the organic soil, while Cr (72.9%) and Fe (20.1%) accrued in the mineral soil. The 6th factor (which is the last factor) revealed that Cu (15.7%) had the highest factor load in the organic and mineral soils relative to all of the studied elements. Cr, Cu, and Pb accumulations in the study probably confirmed intensive pollution from many sources such as industrial, agricultural, commercial, and municipal activities and wastes [28]. These PTEs might have also been deposited during weathering because, in mineral forms, most of the elements are oxidized, released, and reprecipitated in the soil [74]. However, the sources of Mn and Fe in the soil are natural sources, posing no threats if the concentrations do not exceed the maximum allowable limits. However, the sources of Mn and Fe in the soil are natural sources, posing no threats if the concentrations do not exceed the maximum allowable limits. 

### 3.4. Contamination Factor and Pollution Load Index of PTEs for Organic Soil and Mineral Soil 

The research revealed that, in organic soil, there was no obvious pollution recorded in any of the soil sample points except in samples 33, 36, 65, 81, 103, 113, 147, 157, and 193. On the other hand, in the mineral horizon, the soil at some sites were polluted, observed only in samples 111, 126, 213, and 221 horizon (Table S1 and Table S2). This finding, however, is in contrast with the reports from previous studies in the same area. For example, there are studies showing that, in the past few decades, the area was among the major pollution zones caused by industrial and agricultural activities [5,75]. Furthermore, as one of the regions located in northern Bohemia, the study area has been documented as a region characterized by power and coal production from the 1950s to the 1980s [75]. In addition to intensive agriculture, industrial activities, and geological processes, the study area has some peatbogs, and these increased the pollution of the area by PTEs to a large extent [76,77]. This study area lies in the “Black Triangle”, which is affected by industrial activities linked to the extraction and exploitation of coal and other natural resources on the sides of the Jizera Mts areas [20,21,72,73]. The study area is historically susceptible to pollution, but the current results revealed otherwise. This could be due to measures put in place by authorities to ameliorate the impact of industrial activities in the area. 

## 4. Conclusions

In both organic and the mineral soils, a high variability in the PTEs was observed. The spatial distribution and the heterogeneity of the PTEs suggested that there were widely distributed sources of the metals’ enrichment from the industrial, commercial, domestic, and agricultural sectors. Lead revealed a high concentration level. Chromium showed a strong relationship to other elements investigated, while Pb has a significant and strong positive relation with Cu. This was probably because Cr is easily mobilized in acidic soils and our study area is highly acidic.

The correlation between the elements in the same soil layer showed more positive relationships, while the correlation of the PTEs in the mineral soil revealed stronger relationships when compared with the correlation in the organic soil. The findings revealed that all the elements in exemption of Mn indicated significant relationships in both the organic soil and the mineral soil. Meanwhile, the concentrations of Mn and Fe were not harmful in the study area. On the other hand, Mn and Fe we below the EU and World limits. In both the organic soil and the mineral soil, the northern part of the kriged maps revealed more distribution of the elements when compared with either the southern part. This implied that the concentrations of the elements were oriented towards the northern part of the study region.

The applications of the Positive Matrix Factorization (PMF) model, ArcGIS-based ordinary kriging, and contamination level analysis were effective for source identification, hotspot location, and assessment of the contamination level of the PTEs. In the organic soil, there was no obvious pollution recorded in any of the soil sample points except in samples 33, 36, 65, 81, 103, 113, 147, 157, and 193. On the other hand, in the mineral horizon, some deteriorated site quality was observed only in samples 111, 126, 213, and 221. The method and results presented might be applicable in coniferous and broad-leaf tree-dominated highlands with a history of industrial activities and atmospheric acidifications. The methods are suitable for measuring the distribution and concentration of elements.

The current result revealed that there is no evidence of pollution by PTEs in the Jizera Mts area. In contrast, this area lies within the “Black Triangle”, which was affected by industrial activities linked with the extraction and exploitation of coal and other natural resources in Central Europe. However, the rate of pollution in the area is very low based on the findings of this study. There may be a need for intermittent assessment of the soil. This regular assessment will help to curtail the possibility of any excessive accumulation and escalation in the future. The findings from this study may serve as a baseline for pollution assessments of farmland and forest soil quality in the Czech Republic and in Europe. The results might support policy-developers in sustainable farming and forestry for the health of the ecosystem to achieve food security, forest safety, as well as animal and human welfare.

## Figures and Tables

**Table 1:** Mean values of the physiochemical characteristics of the soil in the study area.

Properties (Unit)	Organic Soil	Mineral Soil
Sand (%)	29.7	28.2
Silt (%)	44.2	25.3
Clay (%)	26.1	46.5
Texture	Sandy clay-loam	Clay-loam
N (%)	1.6	0.5
C (%)	30.9	7.5
S (%)	0.34	0.26
P (mg kg^−1^)	946.9	386.2
K (mg kg^−1^)	811.6	935.3
Ca (mg kg^−1^)	915.2	327.9
Mg (mg kg^−1^)	839.5	1078.1
Al (mg kg^−1^)	9473.5	8614.4
pH	3.6	3.8

**Table 2:** Basic statistical characteristics of the soil PTE concentrations in the study area.

Soil Horizons	Parameters ^†^	Cr	Cu	Fe ^‡^	Mn ^‡^	Pb
Organic soil	Count	221	221	221	221	221
	Mean	11.0	16.2	7357.8	149.6	99.2
	Median	9.1	15.5	7010	73	92.9
	Mode	7.2	18.5	10,200	32	104
	Minimum	3.1	2.3	1004	1.0	7.1
	Maximum	85.2	81.9	21,000	1650	339
	Std dev	10.6	8.9	3407.5	145.2	49
	Coef of Var. (CV)	96.36	54.94	46.31	97.06	49.4
Mineral soil	Count	221	221	221	221	221
	Mean	4.5	6.4	6744.3	168.0	65.6
	Median	3.8	3.8	6194.4	68.4	58.8
	Mode	3.9	1.0	3610	248	111
	Minimum	0.4	0.2	159.3	0.5	6.7
	Maximum	26.5	38.3	24,274.0	1940.0	281.0
	Std dev	3.0	6.2	4054.5	137.6	42.3
	Coef of Var. (CV) * Czech Republic	66.7 <11.0	96.88 <16.0	60.12 >8000	81.9 <150.0	64.48 <60.0
	** European mean value	94.8	17.3	38,000	524	32
	** World mean value	59.5	38.9	35,000	488	27
	** Crati Basin	90.54	44.36	54,700	1300	63.67

* Authors’ estimates from most publications in the Czech Republic on the issue as there was no official existing baseline; ** Kabata-Pendias [10]. ^†^ All parameters and numbers are reported in mg kg^−1^, while CV is reported in %. **^‡^** Fe and Mn also showed reasonable variability; they posed no threat because their concentrations were far below the EU and world limits.

**Table 3:** Summary of correlation analyses between the PTE concentrations in the organic and mineral soils.

Parameters	Cr_tot_org	Cu_tot_org	Fe_tot_org	Mn_tot_org	Pb_tot_org	Cr_tot_A	Cu_tot_A	Fe_tot_A	Mn_tot_A	Pb_tot_A
Cr_tot_org	1.00									
Cu_tot_org	0.03	1.00								
Fe_tot_org	0.77 *	0.43 *	1.00							
Mn_tot_org	0.52 **	−0.40	0.19	1.00						
Pb_tot_org	0.00	0.71 **	0.64 *	−0.56 *	1.00					
Cr_tot_A	0.53 *	−0.14	0.10	0.58	−0.21	1.00				
Cu_tot_A	0.10	−0.13	−0.10	0.20	−0.26 *	0.78 *	1.00			
Fe_tot_A	0.03	0.16	0.50 *	−0.06	0.43	0.56 **	0.00	1.00		
Mn_tot_A	0.10	−0.22 *	−0.05	0.73 *	−0.59 **	0.60 *	0.58 *	0.03	1.00	
Pb_tot_A	0.05	−0.12	−0.08	0.10	−0.10	0.76 **	0.81 **	0.19	0.54 *	1.00

* = Correlation is significant at the 0.01 *p*-value, at <0.05; ** = correlation is significant at the 0.05 *p*-value; tot_org = total concentration in organic soil; tot_A = total concentration in the mineral soil.

**Table 4:** Summary of ANOVA for PTE concentrations for the tot-org and tot-A horizons.

Soil Parameters	df	F-Statistics	*p*-Value *
Cr_tot_org	220	2.12	0.019
Cu_tot_org	220	−3.73	<0.001
Fe_tot_org	220	−1.40	<0.001
Mn_tot_org	220	3.31	0.685
Pb_tot_org	220	0.63	<0.001
Cr_tot_A	220	2.06	0.016
Cu_tot_A	220	−1.94	0.021
Fe_tot_A	220	−1.13	<0.001
Mn_tot_A	220	4.91	0.283
Pb_tot_A	220	0.82	0.041

* Figures (or values) in bold are significant at the 0.05 confidence level. tot_org = total concentration in organic soil; tot_A = total concentration in the mineral soil.

## Data Availability

The data are contained within the article and Supplementary Materials.

## Supplementary Materials

The following are available online at https://www.mdpi.com/article/10.3390/toxics9080181/s1, Table S1: Contamination Factor (CF) and Pollution Loading Index (PLI) for PTEs in organic soil (*n* = 221), Table S2: Contamination Factor (CF) and Pollution Loading Index (PLI) for PTEs in mineral soil (*n* = 221).
