# Supplementary material for: Modeling and Assessing the Spatial and Vertical Distributions of Potentially Toxic Elements in Soil and How the Concentrations Differ
Source: Toxics. 2021 Jul 31;9(8):181. doi: 10.3390/toxics9080181 (PMC8402386; doi:10.3390/toxics9080181)
Supplement: Supplementary file 1 [file toxics-09-00181-s001.zip › toxics-1200740-supplementary.pdf]

toxics

MDPI

Article

Modeling and Assessing the Spatial and Vertical Distributions of Potentially Toxic Elements in Soil and How the Concentrations Differ

Samuel Kudjo Ahado, Chukwudi Nwaogu, Vincent Yaw Oppong Sarkodie and Luboš Borůvka

Table S1. Contamination Factor (CF) and Pollution Loading Index (PLI) for PTEs in the organic soil horizon (*N* = 221).

Sample	CF_Cr	CF_Cu	CF_Pb	PLI	Interpretation/Significance
1	0.13	0.47	2.13	0.51	Non- polluted
2	0.09	0.16	1.41	0.27	Non- polluted
3	0.11	0.35	2.37	0.45	Non- polluted
4	0.11	0.17	1.97	0.34	Non- polluted
5	0.11	0.28	3.85	0.49	Non- polluted
6	0.12	0.25	2.69	0.43	Non- polluted
7	0.10	0.22	2.10	0.35	Non- polluted
8	0.10	0.31	3.26	0.47	Non- polluted
9	0.10	0.20	2.48	0.37	Non- polluted
10	0.08	0.19	2.06	0.31	Non- polluted
11	0.06	0.07	0.68	0.14	Non- polluted
12	0.21	0.52	3.01	0.69	Non- polluted
13	0.20	0.53	5.37	0.83	Non- polluted
14	0.18	0.47	3.63	0.68	Non- polluted
15	0.20	0.62	3.26	0.74	Non- polluted
16	0.19	0.48	2.61	0.62	Non- polluted
17	0.23	0.43	2.86	0.65	Non- polluted
18	0.21	0.56	1.97	0.61	Non- polluted
19	0.16	0.57	2.24	0.59	Non- polluted
20	0.15	0.48	3.44	0.63	Non- polluted
21	0.19	0.54	4.07	0.76	Non- polluted
22	0.21	0.55	6.67	0.91	Non- polluted
23	0.18	0.58	5.96	0.85	Non- polluted
24	0.20	0.55	5.48	0.84	Non- polluted
25	0.09	0.17	2.36	0.33	Non- polluted
26	0.16	0.58	4.52	0.74	Non- polluted
27	0.23	0.77	4.78	0.94	Non- polluted
28	0.20	0.85	3.89	0.87	Non- polluted
29	0.12	0.37	4.93	0.60	Non- polluted
30	0.15	0.45	6.07	0.74	Non- polluted
31	0.16	0.59	3.89	0.72	Non- polluted
32	0.13	0.39	2.55	0.51	Non- polluted
33	0.19	0.83	7.04	1.03	Deteriorating site quality
34	0.19	0.68	5.22	0.87	Non- polluted
35	0.19	0.53	9.37	0.98	Non- polluted
36	0.19	0.81	12.56	1.24	Deteriorating site quality
37	0.16	0.51	5.81	0.77	Non- polluted
38	0.10	0.48	3.30	0.54	Non- polluted

39	0.14	0.61	5.22	0.76	Non- polluted
40	0.13	0.47	6.59	0.74	Non- polluted
41	0.15	0.59	4.44	0.73	Non- polluted
42	0.11	0.35	2.57	0.46	Non- polluted
43	0.19	0.60	4.56	0.81	Non- polluted
44	0.15	0.48	5.07	0.71	Non- polluted
45	0.12	0.32	2.69	0.47	Non- polluted
46	0.23	0.83	4.56	0.96	Non- polluted
47	0.19	0.70	5.81	0.91	Non- polluted
48	0.12	0.48	2.10	0.49	Non- polluted
49	0.23	0.81	4.81	0.97	Non- polluted
50	0.10	0.46	2.35	0.48	Non- polluted
51	0.05	0.28	1.43	0.27	Non- polluted
52	0.22	0.30	2.04	0.51	Non- polluted
53	0.17	0.62	4.30	0.77	Non- polluted
54	0.19	0.45	3.89	0.69	Non- polluted
55	0.12	0.44	2.98	0.54	Non- polluted
56	0.22	0.72	3.11	0.79	Non- polluted
57	0.08	0.41	1.93	0.40	Non- polluted
58	0.25	0.55	3.70	0.80	Non- polluted
59	0.14	0.34	2.68	0.50	Non- polluted
60	0.13	0.49	3.44	0.61	Non- polluted
61	0.11	0.54	3.54	0.59	Non- polluted
62	0.15	0.50	4.15	0.67	Non- polluted
63	0.15	0.43	4.15	0.64	Non- polluted
64	0.17	0.48	5.63	0.76	Non- polluted
65	0.16	1.35	4.96	1.02	Deteriorating site quality
66	0.20	0.53	3.52	0.72	Non- polluted
67	0.18	0.63	3.27	0.72	Non- polluted
68	0.21	0.65	4.00	0.81	Non- polluted
69	0.25	0.65	4.11	0.87	Non- polluted
70	0.21	0.73	4.48	0.89	Non- polluted
71	0.19	0.52	3.56	0.71	Non- polluted
72	0.24	0.57	4.81	0.87	Non- polluted
73	0.24	0.61	4.19	0.85	Non- polluted
74	0.17	0.62	4.30	0.78	Non- polluted
75	0.15	0.17	1.79	0.35	Non- polluted
76	0.15	0.34	7.19	0.72	Non- polluted
77	0.19	0.53	4.26	0.75	Non- polluted
78	0.18	0.58	4.37	0.77	Non- polluted
79	0.14	0.49	4.00	0.65	Non- polluted
80	0.17	0.51	5.00	0.76	Non- polluted
81	0.21	0.80	5.81	1.00	Baseline level of pollutant present
82	0.19	0.76	5.37	0.92	Non- polluted
83	0.16	0.58	3.93	0.72	Non- polluted
84	0.18	0.56	5.85	0.84	Non- polluted
85	0.17	0.40	6.30	0.75	Non- polluted
86	0.14	0.61	6.78	0.83	Non- polluted
87	0.21	0.61	6.74	0.95	Non- polluted
88	0.18	0.47	6.96	0.84	Non- polluted
89	0.12	0.43	5.07	0.64	Non- polluted

90	0.23	0.76	5.00	0.95	Non- polluted
91	0.21	0.54	4.07	0.77	Non- polluted
92	0.11	0.38	2.64	0.48	Non- polluted
93	0.23	0.52	6.93	0.93	Non- polluted
94	0.11	0.68	2.19	0.55	Non- polluted
95	0.12	0.34	2.09	0.45	Non- polluted
96	0.09	0.27	2.30	0.39	Non- polluted
97	0.19	0.38	4.33	0.68	Non- polluted
98	0.12	0.46	4.26	0.61	Non- polluted
99	0.18	0.35	3.00	0.58	Non- polluted
100	0.13	0.43	3.23	0.56	Non- polluted
101	0.17	0.53	4.22	0.72	Non- polluted
102	0.24	0.48	4.26	0.79	Non- polluted
103	0.29	0.88	5.04	1.09	Deteriorating site quality
104	0.22	0.45	5.15	0.79	Non- polluted
105	0.10	0.23	3.85	0.45	Non- polluted
106	0.12	0.23	4.37	0.50	Non- polluted
107	0.15	0.34	3.50	0.56	Non- polluted
108	0.12	0.22	2.84	0.42	Non- polluted
109	0.71	0.34	3.07	0.90	Non- polluted
110	0.14	0.36	3.10	0.54	Non- polluted
111	0.63	0.29	2.43	0.76	Non- polluted
112	1.18	0.15	1.04	0.57	Non- polluted
113	1.43	0.31	2.86	1.08	Deteriorating site quality
114	1.19	0.31	2.34	0.95	Non- polluted
115	0.08	0.26	1.68	0.33	Non- polluted
116	0.09	0.31	1.51	0.34	Non- polluted
117	0.08	0.28	1.75	0.34	Non- polluted
118	0.08	0.06	0.46	0.13	Non- polluted
119	0.17	0.24	1.29	0.37	Non- polluted
120	0.09	0.16	1.31	0.27	Non- polluted
121	0.11	0.43	4.48	0.61	Non- polluted
122	0.13	0.53	3.68	0.64	Non- polluted
123	0.09	0.16	1.41	0.27	Non- polluted
124	0.12	0.19	1.44	0.32	Non- polluted
125	0.10	0.31	3.26	0.47	Non- polluted
126	0.07	0.26	2.16	0.35	Non- polluted
127	0.08	0.20	1.53	0.29	Non- polluted
128	0.12	0.17	2.63	0.39	Non- polluted
129	0.29	0.33	2.26	0.60	Non- polluted
130	0.15	0.33	3.10	0.53	Non- polluted
131	0.15	0.45	5.19	0.71	Non- polluted
132	0.43	0.21	1.33	0.49	Non- polluted
133	0.18	0.41	3.17	0.61	Non- polluted
134	0.19	0.59	3.74	0.74	Non- polluted
135	0.11	0.24	2.70	0.42	Non- polluted
136	0.18	0.39	3.70	0.64	Non- polluted
137	0.16	0.36	1.88	0.47	Non- polluted
138	0.18	0.48	2.49	0.59	Non- polluted
139	0.17	0.58	3.44	0.70	Non- polluted
140	0.18	0.55	3.27	0.69	Non- polluted

141	0.17	0.54	5.93	0.82	Non- polluted
142	0.12	0.17	3.54	0.41	Non- polluted
143	0.13	0.31	3.78	0.54	Non- polluted
144	0.16	0.43	7.30	0.80	Non- polluted
145	0.15	0.54	6.22	0.79	Non- polluted
146	0.20	0.68	5.93	0.93	Non- polluted
147	0.21	0.66	7.22	1.00	Baseline level of pollutant present
148	0.16	0.22	1.93	0.41	Non- polluted
149	0.22	0.79	5.70	0.99	Non- polluted
150	0.16	0.67	7.15	0.92	Non- polluted
151	0.12	0.40	3.28	0.54	Non- polluted
152	0.17	0.34	3.26	0.57	Non- polluted
153	0.11	0.25	2.57	0.42	Non- polluted
154	0.19	0.70	5.56	0.91	Non- polluted
155	0.15	0.34	3.57	0.57	Non- polluted
156	0.43	0.49	3.43	0.90	Non- polluted
157	0.26	0.88	9.26	1.29	Deteriorating site quality
158	0.18	0.42	4.93	0.72	Non- polluted
159	0.12	0.15	4.04	0.42	Non- polluted
160	0.14	0.18	2.50	0.40	Non- polluted
161	0.17	0.43	6.15	0.77	Non- polluted
162	0.16	0.46	7.74	0.83	Non- polluted
163	0.16	0.29	3.78	0.56	Non- polluted
164	0.13	0.16	4.04	0.43	Non- polluted
165	0.10	0.15	3.51	0.38	Non- polluted
166	0.14	0.50	5.11	0.70	Non- polluted
167	0.16	0.32	5.19	0.64	Non- polluted
168	0.18	0.65	5.41	0.87	Non- polluted
169	0.06	0.29	1.24	0.27	Non- polluted
170	0.18	0.56	4.74	0.78	Non- polluted
171	0.14	0.21	2.39	0.41	Non- polluted
172	0.22	0.47	5.37	0.82	Non- polluted
173	0.40	0.56	3.85	0.96	Non- polluted
174	0.12	0.14	3.52	0.39	Non- polluted
175	0.14	0.21	2.77	0.43	Non- polluted
176	0.13	0.20	4.19	0.48	Non- polluted
177	0.14	0.32	3.63	0.55	Non- polluted
178	0.14	0.28	4.48	0.57	Non- polluted
179	0.13	0.33	2.30	0.46	Non- polluted
180	0.22	0.18	3.24	0.51	Non- polluted
181	0.22	0.33	4.04	0.66	Non- polluted
182	0.12	2.11	2.78	0.89	Non- polluted
183	0.17	0.12	2.59	0.37	Non- polluted
184	0.15	0.20	4.67	0.51	Non- polluted
185	0.17	0.21	2.97	0.47	Non- polluted
186	0.11	0.23	3.45	0.44	Non- polluted
187	0.14	0.26	4.37	0.55	Non- polluted
188	0.16	0.08	1.85	0.29	Non- polluted
189	0.15	0.14	3.41	0.42	Non- polluted
190	0.18	0.36	4.56	0.67	Non- polluted
191	0.14	0.18	2.91	0.42	Non- polluted

192	0.98	0.23	2.17	0.78	Non- polluted
193	0.25	0.40	10.78	1.02	Deteriorating site quality
194	0.16	0.56	4.15	0.72	Non- polluted
195	0.31	0.44	2.01	0.65	Non- polluted
196	0.45	0.13	1.75	0.47	Non- polluted
197	1.30	0.27	1.99	0.89	Non- polluted
198	0.07	0.17	1.19	0.24	Non- polluted
199	0.06	0.06	0.26	0.10	Non- polluted
200	0.09	0.25	3.19	0.42	Non- polluted
201	0.09	0.32	2.69	0.43	Non- polluted
202	0.09	0.26	1.26	0.31	Non- polluted
203	0.08	0.09	1.14	0.20	Non- polluted
204	0.07	0.18	1.95	0.29	Non- polluted
205	0.10	0.40	3.01	0.49	Non- polluted
206	0.11	0.48	3.32	0.55	Non- polluted
207	0.08	0.19	2.39	0.33	Non- polluted
208	0.07	0.21	1.61	0.29	Non- polluted
209	0.07	0.19	1.69	0.28	Non- polluted
210	0.15	0.43	2.86	0.57	Non- polluted
211	0.13	0.30	3.47	0.52	Non- polluted
212	0.11	0.35	2.37	0.45	Non- polluted
213	0.11	0.17	1.97	0.34	Non- polluted
214	0.12	0.25	2.69	0.43	Non- polluted
215	0.10	0.20	2.48	0.37	Non- polluted
216	0.08	0.13	1.63	0.25	Non- polluted
217	0.08	0.19	2.06	0.31	Non- polluted
218	0.06	0.07	0.68	0.14	Non- polluted
219	0.06	0.19	1.23	0.24	Non- polluted
220	0.09	0.40	2.64	0.45	Non- polluted
221	0.13	0.17	1.04	0.29	Non- polluted

Table S2. Contamination Factor (CF) and Pollution Loading Index (PLI) for PTEs in the mineral soil horizon (N = 221).

Sample	CF_Cr	CF_Cu	CF_Pb	PLI	Interpretation/Significance
1	0.03	0.05	0.76	0.10	Non-polluted
2	0.02	0.03	0.43	0.06	Non-polluted
3	0.01	0.01	0.25	0.03	Non-polluted
4	0.04	0.03	0.80	0.10	Non-polluted
5	0.01	0.02	0.46	0.04	Non-polluted
6	0.05	0.03	0.63	0.10	Non-polluted
7	0.03	0.02	0.30	0.05	Non-polluted
8	0.03	0.01	0.60	0.04	Non-polluted
9	0.03	0.03	0.50	0.07	Non-polluted
10	0.01	0.02	0.37	0.04	Non-polluted
11	0.03	0.05	0.77	0.10	Non-polluted
12	0.08	0.06	0.98	0.17	Non-polluted
13	0.08	0.09	1.69	0.23	Non-polluted
14	0.08	0.05	1.14	0.17	Non-polluted
15	0.08	0.06	0.87	0.16	Non-polluted
16	0.06	0.05	1.14	0.15	Non-polluted
17	0.07	0.07	1.21	0.18	Non-polluted
18	0.02	0.02	0.63	0.07	Non-polluted

19	0.01	0.02	0.37	0.05	Non-polluted
20	0.04	0.04	0.93	0.11	Non-polluted
21	0.06	0.04	1.24	0.15	Non-polluted
22	0.11	0.08	2.77	0.29	Non-polluted
23	0.08	0.10	2.55	0.27	Non-polluted
24	0.10	0.07	2.74	0.27	Non-polluted
25	0.03	0.04	1.24	0.12	Non-polluted
26	0.04	0.05	3.53	0.19	Non-polluted
27	0.09	0.17	3.58	0.38	Non-polluted
28	0.04	0.15	3.65	0.28	Non-polluted
29	0.03	0.10	1.78	0.17	Non-polluted
30	0.05	0.10	1.08	0.17	Non-polluted
31	0.08	0.13	2.25	0.28	Non-polluted
32	0.03	0.09	2.61	0.19	Non-polluted
33	0.07	0.07	1.70	0.21	Non-polluted
34	0.07	0.13	1.98	0.25	Non-polluted
35	0.07	0.06	1.46	0.18	Non-polluted
36	0.03	0.06	1.78	0.15	Non-polluted
37	0.06	0.04	2.18	0.17	Non-polluted
38	0.08	0.14	2.27	0.29	Non-polluted
39	0.04	0.12	3.51	0.26	Non-polluted
40	0.06	0.04	2.74	0.19	Non-polluted
41	0.05	0.12	3.55	0.28	Non-polluted
42	0.02	0.09	1.98	0.16	Non-polluted
43	0.06	0.06	1.85	0.19	Non-polluted
44	0.05	0.04	2.25	0.16	Non-polluted
45	0.06	0.12	2.64	0.26	Non-polluted
46	0.06	0.06	3.00	0.23	Non-polluted
47	0.05	0.12	2.78	0.26	Non-polluted
48	0.06	0.04	1.13	0.14	Non-polluted
49	0.16	0.06	1.79	0.25	Non-polluted
50	0.03	0.18	2.36	0.24	Non-polluted
51	0.06	0.15	3.86	0.32	Non-polluted
52	0.12	0.14	2.28	0.34	Non-polluted
53	0.04	0.04	1.18	0.13	Non-polluted
54	0.10	0.13	1.58	0.27	Non-polluted
55	0.07	0.23	1.80	0.31	Non-polluted
56	0.07	0.18	2.27	0.31	Non-polluted
57	0.06	0.23	3.17	0.34	Non-polluted
58	0.14	0.24	4.34	0.53	Non-polluted
59	0.05	0.06	1.69	0.18	Non-polluted
60	0.03	0.17	1.32	0.18	Non-polluted
61	0.14	0.32	3.89	0.56	Non-polluted
62	0.04	0.08	2.21	0.19	Non-polluted
63	0.03	0.10	2.13	0.18	Non-polluted
64	0.07	0.10	2.67	0.27	Non-polluted
65	0.04	0.98	2.41	0.46	Non-polluted
66	0.06	0.08	1.48	0.19	Non-pollutted
67	0.05	0.17	1.73	0.24	Non-pollutted
68	0.04	0.09	2.10	0.21	Non-pollutted
69	0.06	0.09	1.42	0.21	Non-pollutted

70	0.02	0.05	1.30	0.11	Non-pollutted
71	0.04	0.04	1.03	0.12	Non-pollutted
72	0.05	0.10	2.98	0.25	Non-pollutted
73	0.05	0.06	1.69	0.17	Non-pollutted
74	0.04	0.06	2.27	0.17	Non-pollutted
75	0.05	0.05	1.09	0.13	Non-pollutted
76	0.04	0.06	2.30	0.17	Non-pollutted
77	0.08	0.15	2.75	0.32	Non-pollutted
78	0.06	0.16	3.37	0.32	Non-pollutted
79	0.09	0.10	1.22	0.22	Non-pollutted
80	0.12	0.09	2.40	0.30	Non-pollutted
81	0.12	0.26	2.51	0.43	Non-pollutted
82	0.05	0.07	2.14	0.20	Non-pollutted
83	0.06	0.14	2.32	0.27	Non-pollutted
84	0.06	0.08	2.32	0.23	Non-pollutted
85	0.02	0.08	1.87	0.15	Non-pollutted
86	0.03	0.08	2.16	0.17	Non-pollutted
87	0.04	0.07	2.74	0.20	Non-pollutted
88	0.08	0.09	2.24	0.25	Non-pollutted
89	0.06	0.12	2.84	0.27	Non-pollutted
90	0.08	0.17	2.72	0.33	Non-pollutted
91	0.03	0.07	1.69	0.16	Non-pollutted
92	0.06	0.11	2.97	0.27	Non-pollutted
93	0.04	0.09	3.57	0.23	Non-pollutted
94	0.05	0.19	2.82	0.29	Non-pollutted
95	0.08	0.12	1.32	0.23	Non-pollutted
96	0.05	0.09	1.93	0.20	Non-pollutted
97	0.06	0.09	2.34	0.23	Non-pollutted
98	0.06	0.12	2.47	0.26	Non-pollutted
99	0.04	0.22	2.74	0.30	Non-pollutted
100	0.08	0.16	3.06	0.33	Non-pollutted
101	0.04	0.09	1.68	0.18	Non-pollutted
102	0.07	0.12	2.42	0.27	Non-pollutted
103	0.06	0.21	0.89	0.22	Non-pollutted
104	0.08	0.11	2.55	0.28	Non-pollutted
105	0.05	0.03	2.04	0.14	Non-pollutted
106	0.07	0.05	1.85	0.19	Non-pollutted
107	0.08	0.12	2.01	0.26	Non-pollutted
108	0.07	0.19	2.22	0.30	Non-pollutted
109	0.11	0.17	2.20	0.35	Non-pollutted
110	0.14	0.15	0.83	0.26	Non-pollutted
111	0.21	0.84	10.41	1.22	Deteriorating site quality
112	0.12	0.28	2.77	0.45	Non-pollutted
113	0.17	0.29	2.60	0.51	Non-pollutted
114	0.16	0.46	4.11	0.67	Non-pollutted
115	0.10	0.55	3.70	0.60	Non-pollutted
116	0.09	0.32	2.97	0.44	Non-pollutted
117	0.11	0.28	1.84	0.38	Non-pollutted
118	0.08	0.34	3.85	0.48	Non-pollutted
119	0.12	0.31	3.09	0.48	Non-pollutted
120	0.19	0.26	3.32	0.55	Non-pollutted

121	0.08	0.26	2.83	0.39	Non-pollutted
122	0.07	0.26	2.49	0.35	Non-pollutted
123	0.10	0.38	4.56	0.55	Non-pollutted
124	0.11	0.38	2.73	0.49	Non-pollutted
125	0.15	0.56	6.33	0.80	Non-pollutted
126	0.24	0.74	8.63	1.15	Deteriorating site quality
127	0.13	0.40	4.41	0.62	Non-pollutted
128	0.05	0.02	1.14	0.10	Non-pollutted
129	0.19	0.09	2.63	0.36	Non-pollutted
130	0.05	0.07	2.55	0.22	Non-pollutted
131	0.06	0.20	1.14	0.24	Non-pollutted
132	0.05	0.17	1.12	0.21	Non-pollutted
133	0.04	0.32	3.81	0.36	Non-pollutted
134	0.07	0.03	1.14	0.13	Non-pollutted
135	0.04	0.05	0.81	0.12	Non-pollutted
136	0.08	0.05	0.93	0.16	Non-pollutted
137	0.05	0.07	1.10	0.16	Non-pollutted
138	0.07	0.08	0.86	0.16	Non-pollutted
139	0.03	0.08	1.51	0.15	Non-pollutted
140	0.05	0.05	1.28	0.15	Non-pollutted
141	0.05	0.03	1.05	0.11	Non-pollutted
142	0.03	0.06	2.26	0.16	Non-pollutted
143	0.05	0.05	3.48	0.20	Non-pollutted
144	0.03	0.03	1.00	0.09	Non-pollutted
145	0.08	0.17	1.52	0.27	Non-pollutted
146	0.04	0.10	1.70	0.19	Non-pollutted
147	0.05	0.05	1.12	0.14	Non-pollutted
148	0.03	0.04	1.15	0.10	Non-pollutted
149	0.07	0.07	1.80	0.21	Non-pollutted
150	0.08	0.05	2.54	0.21	Non-pollutted
151	0.09	0.10	1.46	0.23	Non-pollutted
152	0.06	0.05	1.70	0.17	Non-pollutted
153	0.07	0.25	4.67	0.43	Non-pollutted
154	0.04	0.04	2.04	0.15	Non-pollutted
155	0.08	0.08	1.98	0.23	Non-pollutted
156	0.44	0.12	1.51	0.43	Non-pollutted
157	0.05	0.30	5.63	0.43	Non-pollutted
158	0.07	0.04	2.24	0.18	Non-pollutted
159	0.06	0.06	3.03	0.23	Non-pollutted
160	0.05	0.09	1.81	0.19	Non-pollutted
161	0.05	0.14	3.93	0.31	Non-pollutted
162	0.03	0.07	2.72	0.19	Non-pollutted
163	0.04	0.07	2.59	0.20	Non-pollutted
164	0.07	0.04	0.70	0.12	Non-pollutted
165	0.02	0.03	1.11	0.09	Non-pollutted
166	0.06	0.08	2.73	0.23	Non-pollutted
167	0.08	0.07	1.63	0.21	Non-pollutted
168	0.07	0.10	3.62	0.29	Non-pollutted
169	0.06	0.13	2.96	0.28	Non-pollutted
170	0.07	0.14	2.95	0.31	Non-pollutted
171	0.05	0.05	1.56	0.16	Non-pollutted

172	0.09	0.13	1.48	0.25	Non-pollutted
173	0.10	0.12	2.37	0.30	Non-pollutted
174	0.03	0.04	2.74	0.15	Non-pollutted
175	0.10	0.06	2.12	0.23	Non-pollutted
176	0.06	0.13	3.13	0.28	Non-pollutted
177	0.06	0.17	1.97	0.27	Non-pollutted
178	0.04	0.06	1.83	0.16	Non-pollutted
179	0.04	0.12	3.03	0.25	Non-pollutted
180	0.08	0.07	1.51	0.20	Non-pollutted
181	0.09	0.05	1.41	0.19	Non-pollutted
182	0.09	0.03	1.37	0.15	Non-pollutted
183	0.10	0.02	0.40	0.10	Non-pollutted
184	0.10	0.02	1.01	0.13	Non-pollutted
185	0.07	0.05	1.24	0.16	Non-pollutted
186	0.05	0.06	1.54	0.17	Non-pollutted
187	0.04	0.14	2.00	0.23	Non-pollutted
188	0.06	0.09	1.18	0.19	Non-pollutted
189	0.09	0.06	1.58	0.20	Non-pollutted
190	0.12	0.05	1.96	0.24	Non-pollutted
191	0.06	0.22	1.60	0.28	Non-pollutted
192	0.03	0.16	0.78	0.15	Non-pollutted
193	0.10	0.56	4.48	0.62	Non-pollutted
194	0.18	0.55	5.15	0.80	Non-pollutted
195	0.09	0.39	4.11	0.52	Non-pollutted
196	0.13	0.42	5.41	0.67	Non-pollutted
197	0.14	0.34	3.13	0.53	Non-pollutted
198	0.10	0.39	4.04	0.55	Non-pollutted
199	0.10	0.17	1.39	0.29	Non-pollutted
200	0.16	0.74	6.30	0.91	Non-pollutted
201	0.08	0.39	4.11	0.51	Non-pollutted
202	0.09	0.29	2.63	0.41	Non-pollutted
203	0.08	0.32	3.29	0.44	Non-pollutted
204	0.05	0.25	2.20	0.30	Non-pollutted
205	0.05	0.23	1.64	0.26	Non-pollutted
206	0.10	0.22	2.50	0.38	Non-pollutted
207	0.09	0.24	2.10	0.36	Non-pollutted
208	0.06	0.33	3.29	0.41	Non-pollutted
209	0.08	0.27	1.93	0.35	Non-pollutted
210	0.07	0.24	2.17	0.34	Non-pollutted
211	0.08	0.28	2.19	0.37	Non-pollutted
212	0.05	0.21	1.68	0.27	Non-pollutted
213	0.30	0.86	8.85	1.32	Deteriorating site quality
214	0.19	0.35	3.81	0.63	Non-pollutted
215	0.14	0.44	6.67	0.75	Non-pollutted
216	0.16	0.53	6.48	0.82	Non-pollutted
217	0.14	0.49	5.15	0.71	Non-pollutted
218	0.12	0.45	5.22	0.65	Non-pollutted
219	0.13	0.51	6.30	0.76	Non-pollutted
220	0.14	0.53	6.81	0.80	Non-pollutted
221	0.24	0.81	8.56	1.18	Deteriorating site quality
